# Gene Therapy for Parkinson's Disease

**DOI:** 10.1155/2012/757305

**Published:** 2012-03-25

**Authors:** Rachel Denyer, Michael R. Douglas

**Affiliations:** ^1^Department of Neurology, University Hospitals Birmingham NHS Foundation Trust, Mindelson Way, Birmingham B15 2WB, UK; ^2^School of Clinical and Experimental Medicine, University of Birmingham, Edgbaston, Birmingham B15 2TT, UK; ^3^Department of Neurology, Russells Hall Hospital, Dudley Group of Hospitals NHS Foundation Trust, Pensnett Road, Dudley, DY1 2HQ, UK

## Abstract

Current pharmacological and surgical treatments for Parkinson's disease offer symptomatic improvements to those suffering from this incurable degenerative neurological disorder, but none of these has convincingly shown effects on disease progression. Novel approaches based on gene therapy have several potential advantages over conventional treatment modalities. These could be used to provide more consistent dopamine supplementation, potentially providing superior symptomatic relief with fewer side effects. More radically, gene therapy could be used to correct the imbalances in basal ganglia circuitry associated with the symptoms of Parkinson's disease, or to preserve or restore dopaminergic neurons lost during the disease process itself. The latter neuroprotective approach is the most exciting, as it could theoretically be disease modifying rather than simply symptom alleviating. Gene therapy agents using these approaches are currently making the transition from the laboratory to the bedside. This paper summarises the theoretical approaches to gene therapy for Parkinson's disease and the findings of clinical trials in this rapidly changing field.

## 1. Introduction

Parkinson's disease (PD) is a common neurodegenerative disorder that will assume increasing clinical importance in an ageing society, with an average age of onset between 60 and 65 years, but a peak incidence is found between the ages of 70 and 79 years. The specific incidence is dependent on the age structure of the population studied and is difficult to assess precisely but is around 17 per 100,000 according to a systematic review in this area [1].

PD is classically characterised by the loss of striatal dopaminergic neurons within the basal ganglia; however, the underlying pathophysiology is very complex. Both excitatory glutamatergic and inhibitory *γ*-aminobutyric acid pathways (GABA) involved in basal ganglia regulation of movement are affected [2]. These changes lead to disinhibition of subthalamic nucleus (STN) output, which in turn increases the activity of excitatory projections to the internal globus pallidus (GPi) and substantia nigra pars reticularis (SNpr). The net result is increased inhibitory outflow from the GPi and SNpr to other basal ganglia nuclei, thalamus, and cortex, leading to the typical motor features of PD [3]. Various therapeutic approaches that target the STN or GPi have been used to improve motor function in PD, including stereotactic lesioning [4, 5], high frequency deep brain stimulation [6, 7] and pharmacological silencing [8].

Dopamine replacement therapies, such as levodopa, were developed around fifty years ago and still constitute the mainstay of treatment for PD [9]. Patients generally respond very well to this strategy initially, to the extent that failure to respond to levodopa treatment should cause the physician to question the veracity of the diagnosis. However, with long-term treatment, the response to dopamine replacement fluctuates and “wearing off” phenomena or troubling dyskinesias develop [10]. The Parkinson Study Group estimate that over half of patients with early PD receiving levodopa develop at least one of these side effects during the first two years of treatment [10].

The problems with long-term levodopa treatment have led to the search for new therapeutic strategies for PD. Pharmacological agents such as dopamine agonists can be used to delay the initiation of levodopa or as adjuvant therapies. Similarly, catechol O-methyltransferase (COMT) inhibitors are used in an adjuvant role. In more advanced disease, continuous subcutaneous infusions of apomorphine or intraduodenally administered levodopa (Duodopa) can, to some extent, address the problem of fluctuations in clinical response and improve PD symptom control.

The pharmacological and surgical therapies described above aim to improve the symptoms of PD but none are proven to have a significant impact on the underlying disease process with respect to either slowing disease progression or restoring the affected dopaminergic neurons. Gene therapy has distinct potential advantages over conventional treatment modalities for PD as it could theoretically be used to preserve or restore dopaminergic neurons affected by PD through the action of neurotrophic factors [11, 12] or alternatively increase the availability of enzymes required for dopamine synthesis [13, 14]. Although the disease modifying properties of these therapies remain to be proven, they could potentially target the underlying pathophysiological imbalances and may result in much less fluctuation in response and a lower prevalence of dyskinesias than conventional pharmacotherapy for PD. It should be highlighted that PD manifests with additional clinical and paraclinical nonmotor features (including sleep disturbance/fatigue, autonomic, gastrointestinal, neuropsychiatric, and sensory symptoms) [15, 16] which are unlikely to result from specific degeneration of the dopaminergic pathways. Alternative therapeutic approaches will be required to address these issues.

## 2. Gene Delivery

The use of gene therapy to treat PD necessitates the use of a suitable method of delivery for the synthesised nucleic acid—viral or nonviral. The choice of vector greatly influences the technique used for delivery, as a peripherally administered vector must be able to cross the blood-brain barrier with an acceptable degree of tissue specificity. Alternatively, the surgical techniques used for deep brain stimulation can be harnessed to deliver the vector directly to a specific brain region.

Nonviral techniques are technically and conceptually more straightforward but are less well suited to treating a chronic neurodegenerative disorder such as PD, due to the short duration of gene expression that is typically achieved. Low transfection rates mean that experiments using nonviral vectors have often used multiple dose regimens [17]. This poses particular problems for translation to human studies if repeated intracerebral injections, with their associated risks, are needed to achieve a meaningful clinical response. This approach may still prove effective, as seen in a recent study using the human glial cell-derived neurotrophic factor (GDNF) gene and a neurotensin polyplex nanoparticle vector in an animal model of PD, finding that a single intracerebral injection of the agent may prove sufficient to induce a biochemical and functional response [18]. Other nonviral vector studies in animal models of PD have incorporated region-specific ligands in order to maximise tissue specificity using intravenous vector administration [17]. For example, one group [19] has used Trojan horse liposomes and a monoclonal antibody to the transferrin receptor to facilitate transport across the blood brain barrier of a peripherally administered therapeutic plasmid containing DNA for GDNF. They also incorporated the gene promoter for tyrosine hydroxylase (TH), a key enzyme in the synthesis of dopamine, to restrict expression of the transgene to catecholaminergic neurons.

Viral vectors, derived from either DNA or RNA viral vectors, are generally considered to be a more practical approach, with the potential to cause long lasting gene expression via episome formation or DNA integration into the host genome. A range of different types of viruses, each with different properties and advantages, have been exploited in the search for a suitable vector for gene therapy in PD. These are detailed below, with particular attention to adeno-associated viruses which comprise by far the largest category of vectors used in clinical trials to date.

## 3. AAV

Adeno-associated viruses are relatively simple 4.7 Kb single-stranded DNA viruses from the *Parvoviridae* family [20]. They comprise two genes encoding capsid (*cap*) and viral replication (*rep*) proteins and inverted terminal repeat sequences, but require additional genes from other viruses (e.g., adenovirus) for replication. AAVs are well suited to gene therapy for PD as they are capable of inducing long-term gene expression, usually via episome formation [21]. AAVs are also able to integrate into a specific site at chromosome 19 of the human genome raising potential concerns regarding insertional mutagenesis, although the frequency with which integration occurs *in vivo* remains unclear [22, 23]. More than 100 AAV variants have been identified and they are classified into nine genetic clades with differing tissue tropisms [24]. AAVs 1–10 have been used for gene therapy vector production but AAV-2-derived vectors are the best characterised and most frequently utilised serotype in PD gene therapy studies.

One advantage of AAV-2, when administered locally, is that it transduces only neurons within the central nervous system and is particularly efficient in brain regions known to be involved in the pathophysiology of PD, such as the globus pallidus and substantia nigra [22]. A recent rodent study using AAV-2 as a viral vector found that following stereotactic parenchymal injection, 97% of transgene expression was restricted to the targeted subthalamic nucleus and no AAV genomes were detected in recipient blood or cerebrospinal fluid, though a small minority of animals had detectable AAV genomes in nonbrain tissue [25].

Recent research additionally suggests that AAV-1, -5, and -8 are also able to transfect basal ganglia neurons in a highly efficient and specific manner in nonhuman primates and therefore these serotypes could be used in future gene therapy trials [26]. In addition, a recent study investigating the use of erythropoietin as a therapeutic agent for PD successfully delivered the gene to striatal neurons using AAV-9 in a rodent model (discussed below) [27].

One disadvantage of AAV is that approximately 80% of humans exhibit antibodies to AAV-2, which potentially adversely affects AAV-2-mediated gene transfer—particularly outside the CNS. In contrast, the utility of AAV-5 as a viral vector appears to be unaffected by the humoral immune response [28, 29]. It therefore seems likely that future human trials of gene therapy for PD will not be limited to the AAV-2 serotype. There is also evidence for a cellular immune response, which may also have implications for the efficiency of transgene expression using AAV [30]. Another potential problem inherent in using AAVs as vectors is the relatively small size, limiting capacity for inserted DNA to around 4 kb.

**Figure 1:**
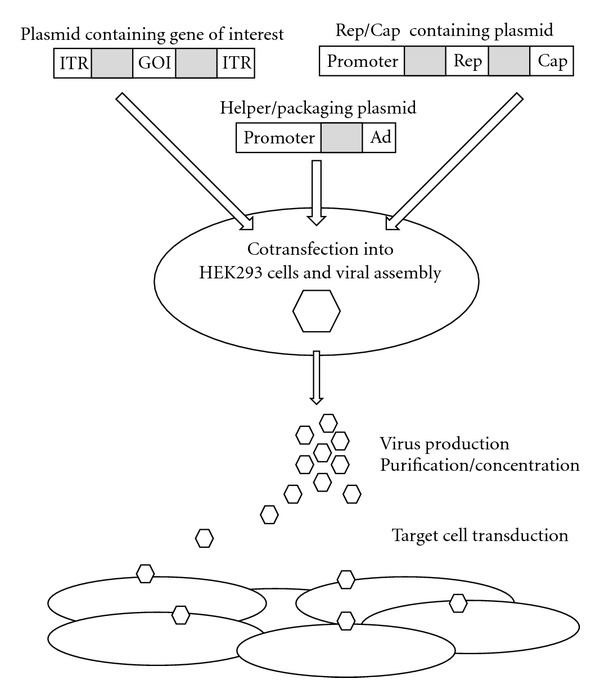
Triple transfection strategy for the *in vitro* production of recombinant adenoassociated viruses (AAV).

AAVs have a very attractive profile in terms of safety, in addition to their tropism for basal ganglia neurons. AAV is not associated with any human disease and the wild-type virus is replication defective [31, 32]. Three-plasmid systems are now well established and routinely used to produce highly purified AAVs, further improving their safety [33] (Figure 1).

## 4. Lentivirus

Lentiviruses are retroviruses that can efficiently infect dividing and nondividing cells [22]. This class includes the human immunodeficiency virus (HIV), which has been studied extensively, and most lentiviral vectors are consequently based on HIV [34]. HIV-1-derived vectors incorporate a transgene between the long terminal repeats (LTRs) required for integration into the host genome. The HIV-1 *env *gene product largely restricts the tropism of wild-type virus to CD4 containing cells. By substituting *env* for gene encoding other viral glycoproteins, such as the vesicular stomatitis virus glycoprotein, the cellular tropism can be broadened or made more specific to neurons [35, 36]. Specificity for neurons can be further improved by the use of specific promoters such as neuron-specific enolase or synapsin-1, while introduction of the human glial fibrillary acidic promoter increases specificity for glia [37, 38].

The more recent two or three plasmid systems have increased the safety profile of lentiviral-based vectors but concerns remain regarding the possibility of recombination events producing a replication competent virus [39]. However, the capacity of these vectors, of approximately 9 kb, makes them a very attractive option for future gene therapy research [40].

## 5. Adenovirus

Adenovirus was one of the first viral vectors used successfully in animal models of PD and contains a 36 kb genome comprised of double-stranded DNA [22, 41]. Wild-type adenovirus is frequently associated with mild respiratory tract, gastrointestinal, and conjunctival infections in humans, though rarer sequelae such as severe adenovirus pneumonia can have a mortality exceeding 50% [42]. Early adenoviral vectors were created using E1 or E3/E4 gene region deletions, but these proved unsatisfactory due to the host inflammatory response and associated toxicity that occurred *in vivo* when the remaining wild-type genes were expressed in the host [43].

Newer “gutless” adenoviral vectors have retained only the inverted terminal repeats from the original wild-type viral genome. They therefore have higher capacity and have been shown to achieve transgene expression with reduced toxicity [44]. However, this appears to have been achieved at the expense of lower transduction efficiency [45] and some innate immune responses to the “gutless” adenoviral vectors persist [46]. Some advantages of this group include the relative ease of scaling up production, in comparison to AAV, for example, and robust gene expression [35, 47].

## 6. HSV

Herpes simplex virus is a 150 kb double-stranded DNA virus, which as a result of its size has a far larger packaging capacity than the viruses already described. In addition to a long-lasting episomal latency, the viral vector is neurotropic—the wild-type virus is associated with encephalitis, as well as cold sores and corneal ulceration [48], and HSV-1 has been shown to infect neurons [49]. HSV-1 vectors can be subdivided into recombinant viral and amplicon vector systems. Recombinant viral systems retain most of the wild-type genome, using homologous recombination to insert the required foreign gene. This system allows for very large genes to be inserted if all the wild-type genes were removed [22]. The amplicon vector, in contrast, contains only a *cis* acting viral origin of replication and packaging signal, with the genes required for replication and virus production are supplied in *trans* by a separate helper virus.

Animal studies suggest that long lasting transgene expression in neurons can be achieved using HSV-1 amplicons and the use of specific promoters, for example, those for tyrosine hydroxylase, can increase transduction specificity [50–52]. There remain some concerns over safety and toxicity of HSV-derived vectors, but research to address these issues is ongoing [35, 53].

## 7. Gene Therapy for Parkinson's Disease

Several complex and interrelated issues need to be addressed in attempting to bring gene therapy for PD from the laboratory to the bedside. The most fundamental of these issues is the selection of a suitable therapeutic target; PD has a complex pathophysiology that is by no means fully understood and involves multiple brain structures and signalling pathways. There are three broad approaches to selection of a therapeutic target. The first and most straightforward of these is to increase dopamine levels in the basal ganglia by the introduction of transgenes encoding enzymes or cell signalling proteins involved in dopamine production or regulation. The key enzymes involved in dopamine metabolism are tyrosine hydroxylase (TH), aromatic amino acid decarboxylase (AADC), and GTP-cyclohydrolase-1 (GCH-1). Animal studies using this approach have been promising and human phase I/II trials of a lentiviral vector containing genes encoding all three key enzymes (ProSavin), are ongoing [14, 54, 55]. The second approach aims to modulate basal ganglia circuitry affected by PD, for example, by increasing levels of GABA to counteract the overactivity of the subthalamic nucleus observed in this condition. Both of these approaches, if successful, are likely to result in symptomatic relief for patients rather than an alteration in disease progression.

The final approach to choosing a therapeutic target aims to use neurotrophic factors, such as brain-derived neurotrophic factor (BDNF) [56, 57], glial cell line-derived neurotrophic factor (GDNF) [11, 58] or neurturin [59], to prevent the death of dopaminergic neurons. This third approach could potentially be disease modifying, in addition to any symptomatic benefit obtained. An overview of therapeutic strategies used in clinical trials of gene therapy for PD is given in Table 1.

## 8. Therapeutic Targets, Agents, and Approaches for Gene Therapy in PD

### 8.1. Aromatic Amino Acid Decarboxylase

Aromatic amino acid decarboxylase (AADC) is an enzyme responsible for the production of dopamine from endogenous or exogenous levodopa. Patients with PD require increasing doses of exogenous levodopa to control their symptoms as the disease progresses and it has been suggested that AADC activity may be reduced in PD. Therefore increasing the activity of this enzyme using gene therapy may reduce both the symptoms of PD and the amount of levodopa required to control them, perhaps also alleviating the side effects of prolonged levodopa therapy [64]. The validity of this proposed therapeutic approach—targeting decarboxylase deficiency—is not absolutely established, as there is evidence suggesting that dopamine is efficiently decarboxylated in 5-hydroxytryptophan (5-HT) immunoreactive neurons [65]. Similarly, it is quite commonly observed in clinical practice that patients with quite advanced PD still benefit from the use of low individual doses of oral levodopa, which would imply that decarboxylase deficiency is not a major issue.

Nonetheless, several preclinical and clinical studies have been published, producing interesting results. Experiments in rhesus monkeys with hemiparkinsonism induced by 1-methyl-4-phenyl-1,2,3,6-tetrahydropyridine (MPTP) show that basal ganglia injection of AAV-AADC induced increased AADC activity *in vivo* and increased immunostaining for AADC, as well as improving dopamine to levodopa ratios [64]. Improvements in AADC activity, sustained transgene expression, lower levodopa requirements, and improved functional outcomes persist up to 8 years after viral injection [66, 67]. Outcomes from these animals at eight years provide reassurance as to the safety and lasting efficacy of this approach in a primate model of PD [67].

A subsequent phase I study using bilateral intraputaminal AAV-AADC injections in patients with moderate to severe PD found a 30% increase in uptake of an AADC tracer [13]. There was also some improvement in 6-month UPDRS scores (both on and off medication), and 3 participants were able to reduce their maintenance dose of levodopa, although there was no control group in this study and results must therefore be interpreted cautiously.

Subsequently, a second group comprising a further five patients were recruited to the phase I study and treated with a slightly higher dose of AAV-AADC (3 × 10^11^ rather than 9 × 10^10^ vector genomes) [60]. UPDRS scores in both the “on” and “off” states were significantly reduced overall, with UPDRS scores showing a more significant reduction in the high-dose group than in the low-dose group, suggesting a potential dose-response relationship. The patients reported significant reductions in “off” time and a nonsignificant reduction in the levodopa dose required to control symptoms was observed in eight patients. Worryingly, three of the 10 subjects studied in total developed intracerebral haemorrhages. While those conducting the study suggest these relate to the neurosurgical procedure used for vector delivery rather than the AAV-AADC itself, it nonetheless contributes to the perception of increased risk associated with this treatment modality when compared to conventional pharmacological therapies. Two otherwise eligible participants were excluded from the study due to raised antibody titres to AAV, highlighting the concern felt regarding potential immune responses against AAV in this type of clinical trial.

### 8.2. Tricistronic Gene Therapy with AADC, GCH-1, and TH

The *in vivo* chemical synthesis of dopamine begins with the conversion of L-tyrosine to levodopa by TH, and then the levodopa is converted to dopamine by AADC. GCH-1 is a rate limiting enzyme in the synthesis of a cofactor for TH called tetrahydrobiopterin. In PD this synthetic process may be deficient at several different points and replacement of a single enzyme may not be sufficient to achieve a clinical response. This has led to attempts to intervene at multiple levels using a lentiviral vector containing genes encoding all three key enzymes required for dopamine synthesis [14].

An early study using a 6-hydroxydopamine (6-OHDA) treated rat model of PD found that the single lentiviral vector was able to successfully transduce all three enzymes and this led to significant functional improvement in motor asymmetry [14]. A subsequent study in nonhuman primates with MPTP-induced parkinsonism found that the same tricistronic lentiviral vector restored extracellular dopamine levels within the striatum and also corrected functional motor deficits for the following 12 months without inducing dyskinesias [55]. Phase I/II human trials using Prosavin, the aforementioned lentiviral vector containing genes for TH, GCH-1, and AADC, are in progress. Preliminary data from the manufacturer suggest that the safety profile and functional response are encouraging, though peer-reviewed outcome data are not yet available.

### 8.3. GAD

Glutamic acid decarboxylase (GAD) is the key enzyme involved in the synthesis of the inhibitory neurotransmitter GABA from excitatory glutamate. PD is associated with hyperactivity of the subthalamic nucleus as a consequence of reduced activity in inhibitory nigrostriatal projections [68, 69]; therefore delivery of the gene encoding GAD could increase local GABA production within the subthalamic nucleus, restoring equilibrium between these pathways. GAD exists in two genetically distinct isoforms, GAD65 and GAD67, with differing anatomical and subcellular distributions, as well as differing enzymatic properties [70–72].

An early *in vitro* study used two recombinant AAV constructs, encoding GAD65 and GAD67, to transduce CNS cell lines leading to transcription of both genetic isoforms and long-term GAD expression [73]. A subsequent *in vivo* study using rats found that injection of AAV/GAD65 or AAV/GAD67 with green fluorescent protein (GFP) into the STN led to long-term expression in the STN in both cases with cellular distributions as predicted for the different isoforms [74]. Electrophysiological recordings were made during the experiment, with a stimulatory electrode placed in the subthalamic nucleus and microdialysis probes and recording electrodes in the SNpr. These revealed that AAV/GAD65-treated rats showed a fourfold statistically significant increase in GABA release following STN stimulation and single unit recording from SNpr demonstrated a significant shift in electrophysiological responses to STN stimulation in AAV/GAD65-treated rats compared to controls, with a greater proportion of inhibitory responses to STN stimulation in the former group.

In addition, one group of rats in this study were treated with direct STN injection of AAV/GAD65 prior to 6-OHDA lesioning of midbrain dopaminergic circuits in the median forebrain bundle [74]. The animals showed significant improvements in several behavioural measures of dopaminergic deficit and locomotion, but also had increased survival of TH positive dopaminergic neurons, in comparison to controls injected with GFP or saline prior to lesioning. The behavioural improvements with AAV/GAD65 in the 6-OHDA rat model of PD have been replicated in a subsequent study, but this did not provide any further evidence to support a neuroprotective effect of GAD65 transduction on dopaminergic neurons [75].

Further studies used a macaque model of PD with carotid injection of MPTP to induce hemiparkinsonism to study the effects of subsequent STN injection of AAV/GAD in comparison to control GFP injection [76]. They found that AAV/GAD-treated hemiparkinsonian macaques showed significant improvements in clinical measures of parkinsonism over 56 weeks with an associated significant increase in ipsilateral fluorodeoxyglucose positron emission tomography (FDG PET) activity, in comparison to controls. The use of AAV/GAD in this primate study raised no new safety concerns and the vector was able to transduce long-term GAD expression, as confirmed by histological analysis. A recent rodent study using AAV2/GAD has provided further evidence of safety, finding that 97% of vector genomes were restricted to the ipsilateral STN following direct STN infusion of the vector, and there was no discernible detrimental effect on animal health or behaviour [25].

These encouraging results in animal studies led Kaplitt and coworkers to conduct the first clinical trial of gene therapy for an adult neurodegenerative disorder [61]. This open label phase I study used stereotactic unilateral STN injection of an AAV-2 serotype viral vector encoding human GAD65 or GAD67 under control of a cytomegalovirus (CMV) promoter. Twelve patients with PD with Hoen and Yahr stage 3 or greater and significant motor fluctuations were recruited and each received a single injection of 50 *μ*L of viral vector in the most symptomatic hemisphere. As the main aim of the study was to assess safety and tolerability, a range of concentrations of vector between 1 × 10^11^ and 10 × 10^11^ was used. Over a followup period of at least 12 months no adverse events related to the trial intervention were recorded, no patient withdrew from the study, and no patient was lost to follow-up. While this study was neither blinded nor designed to establish efficacy, significant improvements in Unified Parkinson's Disease Rating Scale (UPDRS) motor scores were observed in both the “on” and “off” states, with this effect persisting at 12 months. FDG PET demonstrated significant reduction in thalamic metabolism in treated patients on the ipsilateral side only, with an associated increase in metabolism in the supplementary motor cortex that correlated with improvements clinical outcome measures.

The results of a phase II double-blind randomised controlled trial of AAV2/GAD in 45 patients with advanced levodopa responsive PD have recently been published [62]. The intervention used bilateral stereotactic infusion of AAV2/GAD into the subthalamic nucleus, while participants in the control arm received an elaborate sham surgical procedure. While both patient groups had significantly lower UPDRS motor scores at 6 months, the reduction in motor score was significantly greater in the intervention group than controls. In addition, some secondary outcome measures such as the UPDRS global score, also significantly improved in the intervention group compared to controls. One serious adverse event occurred when a patient in the intervention group developed bowel obstruction, though this was not thought to be related to the intervention. Less serious side effects that were more frequent in the intervention group included headache and nausea. Overall, the phase II trial of AAV2/GAD provides substantial support for both the efficacy and the safety of this approach in patients with PD.

### 8.4. GDNF

Glial cell line-derived neurotrophic factor was first characterised nearly two decades ago as a neurotrophic factor for embryonic rodent midbrain dopaminergic neurons, promoting their survival *in vitro* and increasing dopamine uptake in TH-positive neurons without altering uptake of serotonin or GABA [77]. The authors realised its potential application in the treatment of PD and a subsequent *in vivo* study in a mouse model of PD found that direct injection of GDNF into the substantia nigra or striatum resulted in a relative increase in dopaminergic nerve fibre density and improvements in motor behaviour regardless of whether the GDNF was administered before or after the MPTP used to induce parkinsonism [78].

Related experiments used a replication defective adenovirus (Ad) vector to deliver the gene encoding human GDNF as a direct injection near to rat substantia nigra *in vivo*, prior to lesioning with 6-OHDA [41]. Survival of dopaminergic neurons was significantly increased in those rats treated with the Ad/GDNF vector compared to controls; however, transgene expression for both GDNF and the LacZ promoter (used as a transgene in a control group) was reduced over the four week follow-up period of the study, raising doubts about longer-term efficacy of transgene expression. In addition, all animals treated with the Ad vector had localised reactions at the injection site and this effect was observed in both the Ad/GDNF and the Ad/LacZ groups, suggesting it relates to the vector or injection method rather than choice of transgene. Other studies used a lentivirus vector to deliver the GDNF gene by stereotactic striatal and substantia nigra injection *in vivo* in rhesus monkeys one week prior to MPTP treatment [11]. This resulted in an increase in the number of TH-positive dopaminergic neurons in comparison to controls. In addition, the rhesus monkeys treated with the lentiviral vector encoding GDNF performed better in behavioural outcome measures than controls and demonstrated increased and more symmetrical fluorodopa uptake in the striatum in FDG PET scans. Transgene expression of GDNF using this lentiviral approach was sustained at 8 months and there were no issues with host inflammatory responses.

In 2008, Amsterdam Molecular Therapeutics announced that they had acquired a license from Amgen to develop an AAV-based vector to deliver the GDNF gene as a potential new therapy for PD [63], but no clinical trial results in humans have been published using this approach. Future human trials of gene therapy using GDNF for PD will need to take into account the experience of clinical trials of direct recombinant GDNF infusions in PD. Unfortunately, while the initial open label trials in this area were encouraging, with significant reductions in UPDRS off scores in patients who had received the intervention, the findings were not replicated in a phase I/II double-blind randomised controlled trial and subsequent studies found that the clinical effect on UPDRS scores in the open label study was not sustained a year after treatment withdrawal [79–82]. This highlights the importance of incorporating appropriate control groups and suitable lengths of patient followup into the experimental design, particularly as demonstration of sustained efficacy (over years) will be needed for this approach to become an established clinical therapy.

### 8.5. Neurturin

Neurturin (NTN) is a neurotrophic factor which was noted to both share structural similarities to GDNF and to share the ability of GDNF to promote the survival of dopaminergic neurons *in vitro* [83]. Neurturin was subsequently noted to share receptors and signal transduction pathways with GDNF, providing a putative mechanism for its neurotrophic effect [84]. *In vivo* experimentation in rats found that NTN mRNA is expressed during development in the ventral midbrain and striatum, and in addition can increase survival of mature dopaminergic neurons when injected directly into the substantia nigra prior to lesioning with 6-OHDA [85]. Furthermore, injection of NTN directly into the striatum of intact adult rats led to functional overactivity of nigral dopaminergic neurons [85].

In the MPTP primate model of PD, stereotactic injection into the striatum and substantia nigra of an AAV vector containing a gene encoding NTN four days after induction of parkinsonism resulted in preservation of nigral neurons relative to controls [86]. The rhesus monkeys treated with AAV-NTN (also known as CERE-120) showed an 80–90% reduction in motor impairment from 4 months after treatment to the end of the study at 10 months. Subsequently published data found no adverse effects, such as neurotoxicity, and sustained NTN expression up to 12 months after AAV-NTN treatment [87, 88].

Following these encouraging animal studies, 12 patients with PD for at least 5 years underwent bilateral stereotactic intraputaminal injections of AAV serotype 2-neurturin (AAV2-NTN) in an open-label phase I study [59]. During the procedure and the 1-year follow-up period no serious adverse events occurred; however, one patient developed an air embolus related to the surgical intervention itself. The only adverse events the authors felt could potentially be attributed to the AAV2-NTN were three cases of dyskinesias on medication and one patient who developed hallucinations. However, there were more frequent complications, such as headache occurring in eight patients, which the authors attribute to the surgical intervention. While these are not strictly relevant to the safety of AAV2-NTN, these do need to be taken into consideration when comparing the risks and benefits of gene therapy versus conventional medical management in future efficacy (phase III) trials. Secondary outcome measures of efficacy were encouraging, with significant reductions in off-medication UPDRS motor scores at 12 months after intervention compared to baseline.

The results of a phase II double-blind randomised controlled trial of intraputaminal stereotactic AAV2-NTN injection versus sham surgery was recently published [12]. Thirty-eight patients were randomised to the intervention arm and 20 patients to sham surgery and participants were assessed at three monthly intervals until the final patient had completed one year of followup. Unfortunately, there was no significant difference in the primary outcome measure, the reduction in UPDRS off-medication motor score at 12 months, between the control and intervention groups (*P* = 0.91). However, secondary outcome measures demonstrated significant improvements in the mental subscale of the UPDRS in the “off” state and daily living component in the “on” state of the UPDRS, in addition to significant improvements in the PDQ-39 single index score. None of the secondary outcome measures assessed favoured the control group. There was no significant difference in FDG PET scans at baseline and 12 months between control and intervention groups. While the lack of efficacy in the primary outcome measure is disappointing, a subgroup of patients who were followed up for 18 months had a moderate but significant reduction in UPDRS score at this time point [89]. This perhaps suggests that longer follow-up periods may be required to discern efficacy in the treatment group, particularly if the mechanism of action is neuroprotective.

Thirteen of the 38 patients in the intervention group experienced adverse events, compared with four of the 20 control patients [12]. Two patients in the AAV2-NTN group died during the follow-up period—one suffered a myocardial infarction and the other a pulmonary embolus—neither of which were felt to be directly related to AAV2-NTN. Three patients in the AAV2-NTN group developed tumours compared to two patients in the control group. The tumours in the intervention group comprised one patient with glioblastoma (found in retrospect to be present on baseline imaging studies), one with oesophageal adenocarcinoma and one with adenocarcinoma of the prostate. In all cases biopsy tissue was negative for AAV2-NTN when tested using quantitative PCR. The most common side effects occurring more frequently in the intervention group than the control group were headache and nausea.

Recent postmortem studies using brain tissue from two patients who died from unrelated causes have confirmed that AAV2-NTN gene therapy increased neurturin expression in human participants with PD [90]. However, there were differences in the pattern of protein expression in human postmortem brain tissue as compared to that found in the preclinical primate AAV2-NTN studies. Most notable, perhaps, is the lack of neurturin expression in the cell bodies of substantia nigra pars compacta (SNpc) in human brain, in contrast to the robust expression of neurturin and increased TH immunoreactivity primates also treated with AAV2-NTN. These results may have occurred because the time-frame between intervention with AAV2-NTN and death was too short in the patients whose brain tissue was studied (seven weeks in one case) or because of the inability of the MPTP primate model to accurately reflect the pathophysiological mechanisms that occur in PD. The failure to achieve significant retrograde transport of neurturin from the striatum to the SNpc—in contrast to previous primate studies—was particularly marked. Although this could have resulted from technical differences in tissue processing, it was thought more likely that these resulted from a deficiency of axonal transport of the bioactive agent in the studied PD cases. This phenomenon has been postulated to exist in a range of neurodegenerative disorders (amyotrophic lateral sclerosis and Alzheimer's disease, e.g.), and may not be manifest in animal models of PD. The observed axonal transport deficiency was hypothesized to account for the lack of primary endpoint (12 month) therapeutic efficacy of AAV2-NTN in advanced PD patients. As a consequence, the ongoing phase II trials of this agent include protocols involving higher dosages of AAV2-NTN and/or two injection sites in each SNpc. The clinical consequences of these modified protocols will be of great interest once the trials conclude.

### 8.6. Alternative Strategies

As mentioned above, a recent study made use of an AAV-9 vector to deliver the gene for human erythropoietin (Epo) by direct injection into the striatum of 6-OHDA lesioned rats [27]. The authors found that robust expression of human erythropoietin was present for 10 weeks, but also that nigral dopaminergic cells were protected from 6-OHDA-mediated toxicity and the treated rodents showed associated improvements in behavioural outcomes. This offers a new potential neuroprotective target for gene therapy in PD that clearly warrants further investigation. However, one potential problem also noted in this rodent study is that striatal injection of AAV-9/Epo led to increased numbers of peripheral erythrocytes. If significant polycythaemia were to occur in humans, it could put patients at increased risk of complications, potentially including ischaemic heart disease or stroke.

Our increasing appreciation of genetically defined parkinsonian syndromes [91], particularly found in significant numbers in specific ethnic groups and familial PD patient populations, has led to the concept of developing gene therapeutic approaches designed to correct the effects of disease causing mutations. Both dominant (particularly *LRRK2* associated) and recessive (including *parkin*, *PINK1, *and* DJ-1* associated) forms of PD have been identified and the clinical phenotype characterised in detail.

Mutations in *parkin*—an E3 ubiquitin-protein ligase—result in reduced enzymatic activity of the protein, potentially leading to neuronal cell death in the context of a range of cytotoxic insults. Parkin gene therapy has been developed, using either AAV or lentiviral approaches, showing potential efficacy in the alpha-synuclein overexpression rat and macaque models of PD [92–94] and more recently in an MPTP mouse model of PD [95].

Therapies designed for the other well-characterised forms of recessive PD—resulting from mutations in PINK1 or DJ-1—are at an earlier stage of study and development. It is postulated that the relevant mutations result in loss of function of the protein and current research is principally aimed at characterising the pathological results. In the case of DJ-1 for example, knock-down studies in mice suggest that reduced expression of the protein subjects dopaminergic neurons in the SNpc (which appear to be inherently prone to oxidative stress due to their autonomous pacemaker activity) to further increased oxidative stress [96] and cytotoxicity. In a study examining the effects of AAV-delivered Parkin or DJ-1 on MPTP-lesioned mice, either agent led to increased dopaminergic neuronal survival, but neither agent prevented striatal dopamine depletion [97]. These intriguing findings will require further investigation.

RNA interference (RNAi) techniques also offer promising novel therapeutic strategies for PD. Leucine-rich repeat kinase 2 (LRRK2) mutations are thought to cause 10% of familial PD, in an autosomal dominant fashion, and 2-3% of sporadic PD, likely via gain of function mutations. RNAi techniques could theoretically be used to silence expression of alleles containing the LRRK2 mutation without reducing expression of wild-type allelic LRRK2. A recent study used RNAi *in vitro* to achieve efficient allele-specific targeting of two LRRK2 mutations known to occur in PD, though targeting of a third mutation was much less efficient [98]. It may in the future be possible, and perhaps more effective, to target specific genetic mechanisms associated with PD in a particular individual in this manner, where such genetic defects and predispositions are identifiable.

## 9. Design of Clinical Trials of Gene Therapy in Parkinson's Disease

Several complex interrelated issues have to be addressed in the design of clinical trials of gene therapy for PD. These need to reflect the relative lack of clinical experience with gene therapy in comparison with other treatment modalities and the limited ability of animal models to predict outcomes of gene therapy in humans [89, 99]. Concerns about the safety of trialing gene therapy for PD in humans have led to involved debates as to whether relevant transgenes should be placed under the control of cell specific and/or inducible promoters. At first glance, the theoretical advantages of directed and inducible gene expression would appear obvious, and a number of preclinical experiments have used the TH promoter to direct gene expression to dopaminergic cells. Using this approach is not entirely straightforward, however, requiring additional genetic engineering and evaluation. The larger size of the TH promoter (approximately 2.5 kb in size as compared to ~600 bp for CMV) could theoretically be an issue with AAV-based constructs in which genetic capacity is relatively limited. Furthermore, the effectiveness of a therapy designed to promote cell survival (such as GDNF or neurturin) could be reduced if gene delivery and expression is limited to dopaminergic cells that already dead or dying. It may be more therapeutically effective for genes to be expressed in a broad range of cells, with the agent secreted and acting in a paracrine manner.

The theoretical advantages in using vectors with inducible promoters, typically using specific antibiotic agents (such as tetracycline/doxycycline) to drive expression, are obvious, as their expression could be halted if safety concerns arose in the medium or longer term. At present, there is relatively limited *in vivo* data relating to this approach, in contrast to the large number of patients who have participated in clinical trials of (noninducible) AAV-based gene therapy, for either PD or for other diseases such as Alzheimer's or Canavan disease, without any major safety concerns being raised [12, 59, 62, 100, 101]. This has led some to conclude that the continued use of better-established noninducible AAV-based gene therapies may actually be safer—based on the availability of empirical evidence available—than using novel, less well-evaluated, regulatable promoter mechanisms [102].

PD is a heterogenous condition and most clinical trials have excluded older patients with PD and those with dementia, as well as having inclusion criteria that limit the trials to those with more severe disease. The trial populations used may not be representative of the general population of patients with PD in the community as a whole. In addition, it is clearly important to have a suitable control population who undergo blinding where possible, including sham surgical procedures [99], as some gene therapy trials have demonstrated positive effects in open label phase I studies but no significant benefit in the same outcome during a double-blind controlled phase II study [12, 59].

The choice of outcome measure is also problematic; most studies—which largely aim to treat the dopaminergic deficiency of PD—have used the motor component of the UPDRS score, but this neglects effects on the nonmotor symptoms that are often most problematic in late PD. While the UPDRS is likely to remain the major source of outcome data, some authors have highlighted the utility of collecting additional data to find out about other outcomes valued highly by patients themselves [99]. There is unfortunately no objective biomarker at present that has the validity and reliability to be used as a primary outcome measure, though several studies use FDG PET or SPECT studies as a supplementary outcome measure of dopaminergic system functioning [12, 99].

The design of therapeutic gene and its hypothesised therapeutic mechanism should also have a significant impact on study design. For example, a gene therapy aiming to increase release of dopamine in order to achieve symptomatic improvements for PD patients may have observed efficacy over a much shorter period than a gene therapy strategy hoping to achieve neuroprotection. Furthermore, those gene therapies aimed at preserving greater numbers of dopaminergic neurons or improving their functional ability may have the greatest efficacy in patients with early PD while there are a greater proportion of SNpc dopaminergic neurons still remaining, yet those with early PD have not been included in any of the clinical trials of gene therapy to date. Including those with early PD in trials also poses problems in itself because of the lack of a “gold-standard” diagnostic test, the absence of which may increase the heterogeneity of the study group by unwittingly including those who have an alternative diagnosis. Future studies will need to take great care in selecting patients with a defined and where possible homogenous range of “on” and/or “off” scores—as patients with persistently high “on” scores may not benefit significantly from a therapy designed to address dopaminergic deficiency. Similarly, the very high “off” scores found in some patients may be a manifestation of particularly advanced degenerative disease, which may not be amenable to a neuroprotective gene therapy.

The therapeutic target may also impact upon the choice of outcome measures, depending on which symptoms the therapy intends to alleviate, that is, motor or nonmotor, and whether it aims to slow the rate of progress of the disease. Further difficulties will be experienced during prolonged gene therapy trials; as the clinical condition progresses, it is likely that a proportion of enrolled patients will require escalation of therapies to include DBS or infused therapies, which will make dissecting out the specific therapeutic contribution from gene therapy challenging.

## 10. Discussion

Gene therapy has undergone a renaissance since gene therapy trials were halted temporarily in 1999 when a patient died from multiorgan failure after receiving an adenoviral vector gene therapy for ornithine transcarbamylase deficiency [103]. Subsequent technical advances in vector design and production, such as the three-plasmid system, as well as improved regulatory frameworks and more extensive animal studies have served to increase confidence in clinical trials of gene therapy both in principle and practice. By 2008, over 40 clinical trials of gene therapy had been approved by the US Food and Drug Administration, mostly using AAV serotype 2 [104].

The results of several phase I and II clinical trials using AAV-based gene therapy in PD are available, and clinical trials of one lentiviral agent, Prosavin, are ongoing [12, 59, 61–63]. The safety data from these studies are very encouraging with little evidence of serious adverse effects attributable to the therapeutic agents used. Longer term outcome data from these studies will provide additional valuable data regarding the safety of gene therapy for PD when it becomes available. Efficacy data from the clinical trials of gene therapy for PD have been mixed. Phase I studies using AAV to deliver the genes for GAD and for NTN both observed significant positive effects on UPDRS motor scores in the months following the intervention. Unfortunately, the phase II randomised-controlled trial of AAV-NTN failed to find a significant difference in primary endpoint compared to controls, though there were improvements in some secondary outcome measures [12].

The recently published data from the randomised-controlled phase II trial of AAV-GAD in PD patients demonstrated significant improvement in the primary outcome selected, the reduction in off-medication UPDRS score from baseline to 6 months, in patients undergoing intervention rather than sham surgery control [62]. This was the first successful randomised-controlled trial of gene therapy for a neurological disorder and, in addition to bringing gene therapy for PD a step closer, there are broader implications for other neurological disorders. For example, the AAV-GAD trial serves as proof of principle for gene therapy in central nervous system disorders such as the AAV-based gene therapy trials in Canavan disease, a rare type of leukodystrophy [101].

Gene therapy strategies for the treatment of PD are becoming increasingly more sophisticated and the importance of trial design in this field is readily apparent. This has helped to meet some of the challenges inherent in targeting gene therapy to a degenerative central nervous system disorder, including vector delivery to the appropriate brain region, avoidance of neurotoxicity of and identification a suitable molecular target. Future clinical trials of gene therapy for PD are likely to be have a longer duration of followup, particularly if neuroprotection is the proposed mechanism of action of the transgene, and they would benefit from a broader range of validated outcome measures. In addition to offering a promising new treatment modality for a debilitating and incurable disease, the lessons being learned in the hunt for an effective gene therapy for Parkinson's disease offer insight into the potential of gene therapy as a practical and attainable goal in a range of other neurological disorders.

## Figures and Tables

**Table 1:** An overview of the therapeutic approaches used in current clinical trials of gene therapy for Parkinson's disease.

Therapeutic approach	Vector	Clinical trials
Increased dopamine biosynthesis		
(i) AADC alone	Adeno-associated virus	Phase I [13, 60]
(ii) AADC, TH, and GCH-1	Lentivirus	Phase I/ II in progress [54]
Modulation of excitatory and inhibitory basal ganglia activity		
(i) GAD	Adeno-associated virus	Phase I and II [61, 62]
Neurotrophic support		
(i) GDNF	Adeno-associated virus	License obtained to develop, no published clinical trials [63]
(ii) Neurturin	Adeno-associated virus	Phase I and II [12, 59]

AADC, aromatic amino acid decarboxylase; GAD, glutamic acid decarboxylase; GCH-1, GTP-cyclohydrolase-1; GDNF, glial cell line-derived neurotrophic factor; TH, tyrosine hydroxylase.

## References

[1] Twelves D, Perkins KSM, Counsell C (2003). Systematic review of incidence studies of Parkinson’s disease. *Movement Disorders*.

[2] Brown P, Oliviero A, Mazzone P, Insola A, Tonali P, Di Lazzaro V (2001). Dopamine dependency of oscillations between subthalamic nucleus and pallidum in Parkinson’s disease. *Journal of Neuroscience*.

[3] Wichmann T, DeLong MR (2003). Pathophysiology of Parkinson’s disease: the MPTP primate model of the human disorder. *Annals of the New York Academy of Sciences*.

[4] Alvarez L, Macias R, Guridi J (2001). Dorsal subthalamotomy for Parkinson’s disease. *Movement Disorders*.

[5] Su PC, Tseng HM, Liu HM, Yen RF, Liou HH (2003). Treatment of advanced Parkinson’s disease by subthalamotomy: one-year results. *Movement Disorders*.

[6] Benabid AL, Pollak P, Gao D (1996). Chronic electrical stimulation of the ventralis intermedius nucleus of the thalamus as a treatment of movement disorders. *Journal of Neurosurgery*.

[7] Obeso JA, Olanow CW, Rodriguez-Oroz MC, Krack P, Kumar R, Lang AE (2001). Deep-brain stimulation of the subthalamic nucleus or the pars interna of the globus pallidus in Parkinson’s disease. *New England Journal of Medicine*.

[8] Levy R, Lang AE, Dostrovsky JO (2001). Lidocaine and muscimol microinjections in subthalamic nucleus reverse parkinsonian symptoms. *Brain*.

[9] Fahn S (2006). Levodopa in the treatment of Parkinson's disease. *Journal of Neural Transmission*.

[10] Parkinson Study Group (2000). Pramipexole vs levodopa as initial treatment for Parkinson’s disease: a randomised controlled trial. *Journal of the American Medical Association*.

[11] Kordower JH, Emborg ME, Bloch J (2000). Neurodegeneration prevented by lentiviral vector delivery of GDNF in primate models of Parkinson’s disease. *Science*.

[12] Marks WJ, Bartus RT, Siffert J (2010). Gene delivery of AAV2-neurturin for Parkinson’s disease: a double-blind, randomised, controlled trial. *The Lancet Neurology*.

[13] Eberling JL, Jagust WJ, Christine CW (2008). Results from a phase I safety trial of hAADC gene therapy for Parkinson disease. *Neurology*.

[14] Azzouz M, Martin-Rendon E, Barber RD (2002). Multicistronic lentiviral vector-mediated striatal gene transfer of aromatic L-amino acid decarboxylase, tyrosine hydroxylase, and GTP cyclohydrolase I induces sustained transgene expression, dopamine production, and functional improvement in a rat model of Parkinson’s disease. *Journal of Neuroscience*.

[15] Chaudhuri KR, Healy DG, Schapira AHV (2006). Non-motor symptoms of Parkinson’s disease: diagnosis and management. *Lancet Neurology*.

[16] Fox SH, Brotchie JM, Lang AE (2008). Non-dopaminergic treatments in development for Parkinson’s disease. *The Lancet Neurology*.

[17] Huang R, Han L, Li J (2009). Neuroprotection in a 6-hydroxydopamine-lesioned Parkinson model using lactoferrin-modified nanoparticles. *Journal of Gene Medicine*.

[18] Gonzalez-Barrios JA, Lindahl M, Bannon MJ (2006). Neurotensin polyplex as an efficient carrier for delivering the human GDNF gene into nigral dopamine neurons of hemiparkinsonian rats. *Molecular Therapy*.

[19] Xia CF, Boado RJ, Zhang Y, Chu C, Pardridge WM (2008). Intravenous glial-derived neurotrophic factor gene therapy of experimental Parkinson’s disease with Trojan horse liposomes and a tyrosine hydroxylase promoter. *Journal of Gene Medicine*.

[20] Srivastava A, Lusby EW, Berns KI (1983). Nucleotide sequence and organization of the adeno-associated virus 2 genome. *Journal of Virology*.

[21] Schnepp BC, Clark KR, Klemanski DL, Pacak CA, Johnson PR (2003). Genetic fate of recombinant adeno-associated virus vector genomes in muscle. *Journal of Virology*.

[22] Mandel RJ, Burger C, Snyder RO (2008). Viral vectors for in vivo gene transfer in Parkinson’s disease: properties and clinical grade production. *Experimental Neurology*.

[23] Muzyczka N (1992). Use of adeno-associated virus as a general transduction vector for mammalian cells. *Current Topics in Microbiology and Immunology*.

[24] Gao G, Vandenberghe LH, Alvira MR (2004). Clades of adeno-associated viruses are widely disseminated in human tissues. *Journal of Virology*.

[25] Fitzsimons HL, Riban V, Bland RJ, Wendelken JL, Sapan CV, During MJ (2010). Biodistribution and safety assessment of AAV2-GAD following intrasubthalamic injection in the rat. *Journal of Gene Medicine*.

[26] Dodiya HB, Bjorklund T, Stansell J, Mandel RJ, Kirik D, Kordower JH (2010). Differential transduction following basal ganglia administration of distinct pseudotyped AAV capsid serotypes in nonhuman primates. *Molecular Therapy*.

[27] Xue YQ, Ma BF, Zhao LR (2010). AAV9-mediated erythropoietin gene delivery into the brain protects nigral dopaminergic neurons in a rat model of Parkinson’s disease. *Gene Therapy*.

[28] Peden CS, Burger C, Muzyczka N, Mandel RJ (2004). Circulating anti-wild-type adeno-associated virus type 2 (AAV2) antibodies inhibit recombinant AAV2 (rAAV2)-mediated, but not rAAV5-mediated, gene transfer in the brain. *Journal of Virology*.

[29] Sanftner LM, Suzuki BM, Doroudchi MM (2004). Striatal delivery of rAAV-hAADC to rats with preexisting immunity to AAV. *Molecular Therapy*.

[30] Lowenstein PR (2004). Input virion proteins: cryptic targets of antivector immune responses in preimmunized subjects. *Molecular Therapy*.

[31] Monahan PE, Jooss K, Sands MS (2002). Safety of adeno-associated virus gene therapy vectors: a current evaluation. *Expert Opinion on Drug Safety*.

[32] Tenenbaum L, Lehtonen E, Monahan PE (2003). Evaluation of risks related to the use of adeno-associated virus-based vectors. *Current Gene Therapy*.

[33] Samulski RJ, Chang LS, Shenk T (1989). Helper-free stocks of recombinant adeno-associated viruses: normal integration does not require viral gene expression. *Journal of Virology*.

[34] Vigna E, Naldini L (2000). Lentiviral vectors: excellent tools for experimental gene transfer and promising candidates for gene therapy. *Journal of Gene Medicine*.

[35] Verma IM, Weitzman MD (2005). Gene therapy: twenty-first century medicine. *Annual Review of Biochemistry*.

[36] Naldini L, Blömer U, Gallay P (1996). In vivo gene delivery and stable transduction of nondividing cells by a lentiviral vector. *Science*.

[37] Hioki H, Kameda H, Nakamura H (2007). Efficient gene transduction of neurons by lentivirus with enhanced neuron-specific promoters. *Gene Therapy*.

[38] Jakobsson J, Lundberg C (2006). Lentiviral vectors for use in the central nervous system. *Molecular Therapy*.

[39] Zufferey R, Nagy D, Mandel RJ, Naldini L, Trono D (1997). Multiply attenuated lentiviral vector achieves efficient gene delivery in vivo. *Nature Biotechnology*.

[40] Zhao J, Lever AML (2007). Lentivirus-mediated gene expression. *Methods in Molecular Biology*.

[41] Choi-Lundberg DL, Lin Q, Chang YN (1997). Dopaminergic neurons protected from degeneration by GDNF gene therapy. *Science*.

[42] Lynch JP, Fishbein M, Echavarria M (2011). Adenovirus. *Seminars in Respiratory and Critical Care Medicine*.

[43] Lowenstein PR, Castro MG (2003). Inflammation and adaptive immune responses to adenoviral vectors injected into the brain: peculiarities, mechanisms, and consequences. *Gene Therapy*.

[44] Schiedner G, Morral N, Parks RJ (1998). Genomic DNA transfer with a high-capacity adenovirus vector results in improved in vivo gene expression and decreased toxicity. *Nature Genetics*.

[45] Lowenstein PR, Thomas CE, Umana P (2002). High-capacity, helper-dependent, “gutless” adenoviral vectors for gene transfer into brain. *Methods in Enzymology*.

[46] Muruve DA (2004). The innate immune response to adenovirus vectors. *Human Gene Therapy*.

[47] Kamen A, Henry O (2004). Development and optimization of an adenovirus production process. *Journal of Gene Medicine*.

[48] Carr DJJ, Tomanek L (2006). Herpes simplex virus and the chemokines that mediate the inflammation. *Current Topics in Microbiology and Immunology*.

[49] Fink DJ, Glorioso JC (1997). Engineering herpes simplex virus vectors for gene transfer to neurons. *Nature Medicine*.

[50] Cao H, Zhang GR, Wang X, Kong L, Geller AI (2008). Enhanced nigrostriatal neuron-specific, long-term expression by using neural-specific promoters in combination with targeted gene transfer by modified helper virus-free HSV-1 vector particles. *BMC Neuroscience*.

[51] Rasmussen M, Kong L, Zhang GR (2007). Glutamatergic or GABAergic neuron-specific, long-term expression in neocortical neurons from helper virus-free HSV-1 vectors containing the phosphate-activated glutaminase, vesicular glutamate transporter-1, or glutamic acid decarboxylase promoter. *Brain Research*.

[52] Samaniego LA, Neiderhiser L, DeLuca NA (1998). Persistence and expression of the herpes simplex virus genome in the absence of immediate-early proteins. *Journal of Virology*.

[53] Lilley CE, Branston RH, Coffin RS (2001). Herpes simplex virus vectors for the nervous system. *Current Gene Therapy*.

[54] Oxford BioMedica ProSavin. http://www.oxfordbiomedica.co.uk/page.asp?pageid=29.

[55] Jarraya B, Boulet S, Ralph GS (2009). Dopamine gene therapy for Parkinson’s disease in a nonhuman primate without associated dyskinesia. *Science Translational Medicine*.

[56] Hyman C, Hofer M, Barde YA (1991). BDNF is a neurotrophic factor for dopaminergic neurons of the substantia nigra. *Nature*.

[57] Klein RL, Lewis MH, Muzyczka N, Meyer EM (1999). Prevention of 6-hydroxydopamine-induced rotational behavior by BDNF somatic gene transfer. *Brain Research*.

[58] Björklund A, Kirik D, Rosenblad C, Georgievska B, Lundberg C, Mandel RJ (2000). Towards a neuroprotective gene therapy for Parkinson’s disease: use of adenovirus, AAV and lentivirus vectors for gene transfer of GDNF to the nigrostriatal system in the rat Parkinson model. *Brain Research*.

[59] Marks WJ, Ostrem JL, Verhagen L (2008). Safety and tolerability of intraputaminal delivery of CERE-120 (adeno-associated virus serotype 2-neurturin) to patients with idiopathic Parkinson’s disease: an open-label, phase I trial. *The Lancet Neurology*.

[60] Christine CW, Starr PA, Larson PS (2009). Safety and tolerability of putaminal AADC gene therapy for Parkinson disease. *Neurology*.

[61] Kaplitt MG, Feigin A, Tang C (2007). Safety and tolerability of gene therapy with an adeno-associated virus (AAV) borne GAD gene for Parkinson’s disease: an open label, phase I trial. *The Lancet*.

[62] LeWitt PA, Rezai AR, Leehey MA (2011). AAV2-GAD gene therapy for advanced Parkinson's disease: a double-blind, sham-surgery controlled, randomised trial. *The Lancet Neurology*.

[63] Amsterdam Molecular Therapeutics AMT obtains license to amgen’s GDNF gene to develop treatment for Parkinson’s disease with AMT’s proprietary gene therapy platform. Amsterdam molecular therapeutics. http://www.amtbiopharma.com/news/92/182/AMT-Obtains-License-to-Amgen-s-GDNF-Gene-to-Develop-Treatment-for-Parkinson-s-Disease-with-AMT-s-Proprietary-Gene-Therapy-Platform.html.

[64] Bankiewicz KS, Eberling JL, Kohutnicka M (2000). Convection-enhanced delivery of AAV vector in Parkinsonian monkeys; in vivo detection of gene expression and restoration of dopaminergic function using pro-drug approach. *Experimental Neurology*.

[65] Kitahama K, Ikemoto K, Jouvet A (2009). Aromatic l-amino acid decarboxylase-immunoreactive structures in human midbrain, pons, and medulla. *Journal of Chemical Neuroanatomy*.

[66] Bankiewicz KS, Forsayeth J, Eberling JL (2006). Long-term clinical improvement in MPTP-lesioned primates after gene therapy with AAV-hAADC. *Molecular Therapy*.

[67] Hadaczek P, Eberling JL, Pivirotto P, Bringas J, Forsayeth J, Bankiewicz KS (2010). Eight years of clinical improvement in MPTP-lesioned primates after gene therapy with AAV2-hAADC. *Molecular Therapy*.

[68] Hamani C, Saint-Cyr JA, Fraser J, Kaplitt M, Lozano AM (2004). The subthalamic nucleus in the context of movement disorders. *Brain*.

[69] Bergman H, Wichmann T, DeLong MR (1990). Reversal of experimental Parkinsonism by lesions of the subthalamic nucleus. *Science*.

[70] Bu DF, Erlander MG, Hitz BC (1992). Two human glutamate decarboxylases, 65-kDa GAD and 67-kDa GAD, are each encoded by a single gene. *Proceedings of the National Academy of Sciences of the United States of America*.

[71] Erlander MG, Tillakaratne NJK, Feldblum S, Patel N, Tobin AJ (1991). Two genes encode distinct glutamate decarboxylases. *Neuron*.

[72] Feldblum S, Erlander MG, Tobin AJ (1993). Different distributions of GAD65 and GAD67 mRNAs suggest that the two glutamate decarboxylases play distinctive functional roles. *Journal of Neuroscience Research*.

[73] Mi J, Chatterjee S, Wong KK, Forbes C, Lawless G, Tobin AJ (1999). Recombinant adeno-associated virus (AAV) drives constitutive production of glutamate decarboxylase in neural cell lines. *Journal of Neuroscience Research*.

[74] Luo J, Kaplitt MG, Fitzsimons HL (2002). Subthalamic GAD gene therapy in a Parkinson’s disease rat model. *Science*.

[75] Lee B, Lee H, Nam YR, Oh JH, Cho YH, Chang JW (2005). Enhanced expression of glutamate decarboxylase 65 improves symptoms of rat parkinsonian models. *Gene Therapy*.

[76] Emborg ME, Carbon M, Holden JE (2007). Subthalamic glutamic acid decarboxylase gene therapy: changes in motor function and cortical metabolism. *Journal of Cerebral Blood Flow and Metabolism*.

[77] Lin LFH, Doherty DH, Lile JD, Bektesh S, Collins F (1993). GDNF: a glial cell line-derived neurotrophic factor for midbrain dopaminergic neurons. *Science*.

[78] Tomac A, Lindqvist E, Lin LFH (1995). Protection and repair of the nigrostriatal dopaminergic system by GDNF in vivo. *Nature*.

[79] Gill SS, Patel NK, Hotton GR (2003). Direct brain infusion of glial cell line-derived neurotrophic factor in Parkinson disease. *Nature Medicine*.

[80] Lang AE, Gill S, Patel NK (2006). Randomized controlled trial of intraputamenal glial cell line-derived neurotrophic factor infusion in Parkinson disease. *Annals of Neurology*.

[81] Patel NK, Bunnage M, Plaha P, Svendsen CN, Heywood P, Gill SS (2005). Intraputamenal infusion of glial cell line-derived neurotrophic factor in PD: a two-year outcome study. *Annals of Neurology*.

[82] Slevin JT, Gash DM, Smith CD (2007). Unilateral intraputamenal glial cell line-derived neurotrophic factor in patients with Parkinson disease: response to 1 year of treatment and 1 year of withdrawal. *Journal of Neurosurgery*.

[83] Kotzbauer PT, Lampe PA, Heuckeroth RO (1996). Neurturin, a relative of glial-cell-line-derived neurotrophic factor. *Nature*.

[84] Creedon DJ, Tansey MG, Baloh RH (1997). Neurturin shares receptors and signal transduction pathways with glial cell line-derived neurotrophic factor in sympathetic neurons. *Proceedings of the National Academy of Sciences of the United States of America*.

[85] Horger BA, Nishimura MC, Armanini MP (1998). Neurturin exerts potent actions on survival and function of midbrain dopaminergic neurons. *Journal of Neuroscience*.

[86] Kordower JH, Herzog CD, Dass B (2006). Delivery of neurturin by AAV2 (CERE-120)-mediated gene transfer provides structural and functional neuroprotection and neurorestoration in MPTP-treated monkeys. *Annals of Neurology*.

[87] Herzog CD, Dass B, Gasmi M (2008). Transgene expression, bioactivity, and safety of CERE-120 (AAV2-Neurturin) following delivery to the monkey striatum. *Molecular Therapy*.

[88] Herzog CD, Brown L, Gammon D (2009). Expression, bioactivity, and safety 1 year after adeno-associated viral vector type 2-mediated delivery of neurturin to the monkey nigrostriatal system support CERE-120 for Parkinson’s disease. *Neurosurgery*.

[89] Huddleston DE, Factor SA (2011). Of monkeys and men: analysis of the phase 2 double-blind, sham-surgery controlled, randomized trial of AAV2-neurturin gene therapy for parkinson's disease. *Current Neurology and Neuroscience Reports*.

[90] Bartus RT, Herzog CD, Chu Y (2011). Bioactivity of AAV2-neurturin gene therapy (CERE-120): differences between Parkinson's disease and nonhuman primate brains. *Movement Disorders*.

[91] Douglas MR, Lewthwaite AJ, Nicholl DJ (2007). Genetics of Parkinson’s disease and parkinsonism. *Expert Review of Neurotherapeutics*.

[92] Yamada M, Mizuno Y, Mochizuki H (2005). Parkin gene therapy for *α*-synucleinopathy: a rat model of Parkinson’s disease. *Human Gene Therapy*.

[93] Lo Bianco C, Schneider BL, Bauer M (2004). Lentiviral vector delivery of parkin prevents dopaminergic degeneration in an *α*-synuclein rat model of Parkinson’s disease. *Proceedings of the National Academy of Sciences of the United States of America*.

[94] Yasuda T, Miyachi S, Kitagawa R (2007). Neuronal specificity of *α*-synuclein toxicity and effect of Parkin co-expression in primates. *Neuroscience*.

[95] Yasuda T, Hayakawa H, Nihira T (2011). Parkin-mediated protection of dopaminergic neurons in a chronic MPTP-minipump mouse model of parkinson disease. *Journal of Neuropathology and Experimental Neurology*.

[96] Guzman JN, Sanchez-Padilla J, Wokosin D (2010). Oxidant stress evoked by pacemaking in dopaminergic neurons is attenuated by DJ-1. *Nature*.

[97] Paterna JC, Leng A, Weber E, Feldon J, Büeler H (2007). DJ-1 and parkin modulate dopamine-dependent behavior and inhibit MPTP-induced nigral dopamine neuron loss in mice. *Molecular Therapy*.

[98] de Yñigo-Mojado L, Martín-Ruíz I, Sutherland JD (2011). Efficient Allele-specific targeting of LRRK2 R1441 mutations mediated by RNAi. *PLoS ONE*.

[99] Lewis TB, Standaert DG (2008). Design of clinical trials of gene therapy in Parkinson disease. *Experimental Neurology*.

[100] Mandel RJ (2010). CERE-110, an adeno-associated virus-based gene delivery vector expressing human nerve growth factor for the treatment of Alzheimer’s disease. *Current Opinion in Molecular Therapeutics*.

[101] McPhee SWJ, Janson CG, Li C (2006). Immune responses to AAV in a phase I study for Canavan disease. *Journal of Gene Medicine*.

[102] Kordower JH, Olanow CW (2008). Regulatable promoters and gene therapy for Parkinson’s disease: is the only thing to fear, fear itself?. *Experimental Neurology*.

[103] Somia N, Verma IM (2000). Gene therapy: trials and tribulations. *Nature Reviews Genetics*.

[104] Mueller C, Flotte TR (2008). Clinical gene therapy using recombinant adeno-associated virus vectors. *Gene Therapy*.

